# Almond Waste is a Growing Challenge

**DOI:** 10.1021/acscentsci.3c01458

**Published:** 2023-12-07

**Authors:** Robin Meadows

It is early October, and Christine Gemperle, a second-generation almond grower near Ceres, in the heart of California, has just finished harvesting the orchards she farms with her brother.

The truckloads of almonds the Gemperles produce also generate piles upon piles of byproducts in the form of a shell and a hull—a fuzzy, fruit-like outer layer—that together weigh about two to three times as much as the nut.

**Figure:**
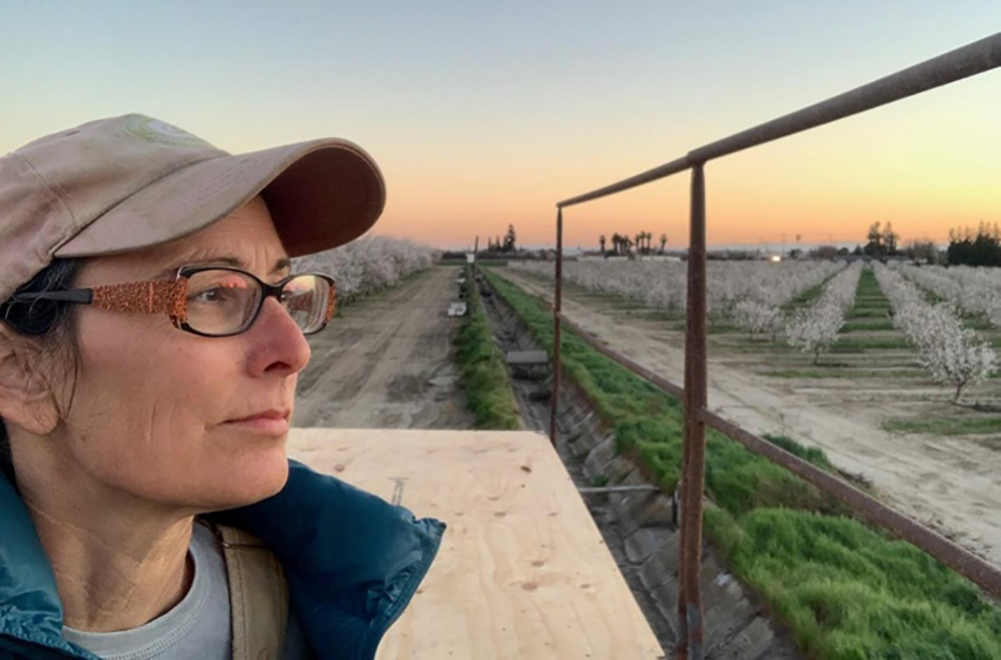
Christine Gemperle overlooks the orchard where she has grown almonds for 25 years. Credit: Courtesy of Christine Gemperle.

California growers produce a total of up to 1.4 million metric tons (t) per year of almonds, which translates into 3 million t of hulls and 1 million t of shells annually. Although nearly all this waste is used, hulls can fetch just $100 per ton, and shells add next to nothing to the industry’s profit.

“We’re trying to change that equation,” says Gemperle, who sits on the Almond Board of California, a trade association that promotes and funds research on the nut. “Almond coproducts could be more beneficial to growers and their bottom line—and also to the environment.”

Hulls are fed to dairy cows, and shells are ground for livestock bedding. But the dairy industry is shrinking in California, where 80% of the world’s almonds are grown. So even though demand for almonds has risen 6-fold in the past 2 decades, demand for the hulls as cattle feed could wane—and the industry is starting to get nervous as their waste keeps piling up.

In response, the almond industry has partnered with researchers in California to get creative about reusing waste. They’re trying to extract more value by folding the sugars and other organic material in almond refuse into commercial products. The hope is that ubiquitous goods such as plastics, rubber, and food could be produced more sustainably. “Consumer preferences are changing, and we want to be ahead of them,” says Danielle Veenstra, the Almond Board’s sustainability lead and a third-generation almond grower.

William Orts, a chemist who is acting director of the U.S. Department of Agriculture Western Regional Research Center, says that there are so many almond shells that “processors sometimes have to pay to get rid of them—the industry is a victim of its own success.”

A decade ago, the almond industry turned to the USDA Bioproducts Research Unit, which Orts led until recently. He is a real-life Rumpelstiltskin. Instead of spinning straw into gold, he transforms agricultural waste into higher-value green products. Displayed proudly in his office are a spoon and a clamshell food container forged from corn-based materials, among other USDA upcycled inventions.

**Figure:**
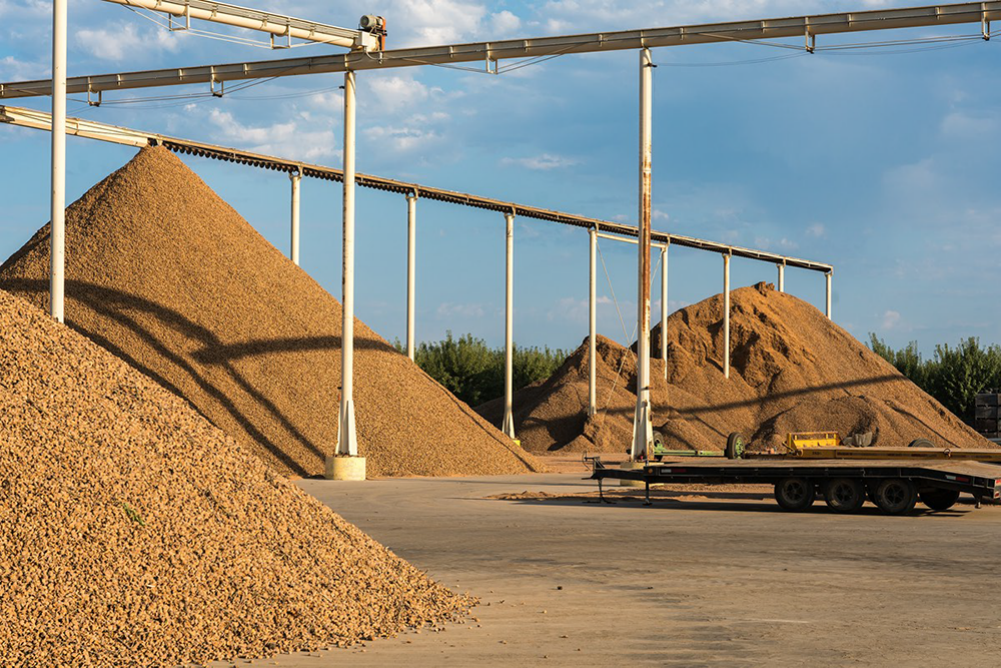
Mounds of almond shells at a processing plant in Modesto, California Credit: Shutterstock.

One of the team’s latest accomplishments leans against a wall in another part of the sprawling research facility: a black plastic shipping pallet. It looks and feels much like any other pallet made from recycled plastic. But this one contains a surprising ingredient: almond shells.

The first step in producing this pallet and other almond waste-based goods was a 3-year investigation into the chemical makeup of hulls and shells. Almonds are in the same family as peaches and apricots, and their fruit-like hulls are similarly rich in fructose and other sugars as well as pectin, a polysaccharide fiber used for thickening jams and jellies. Unlike peach and apricot fruits, however, almond hulls are also full of bitter polyphenols, called tannins, that make them a hard sell as a food ingredient.

Almond shells, the layer under the hull, have a composition similar to that of wood. They consist primarily of three components in roughly equal proportions: cellulose and hemicellulose, which are polysaccharides, and lignin, a phenolic polymer. These compounds add strength and rigidity to plant cell walls.

Once the USDA team knew what they had to work with, they brainstormed new ways to use almond hulls and shells. “That’s when we started to have fun,” Orts recalls. “We said, ‘Let’s see if we can find higher-value, greener uses.’ ” The group’s first question was whether it could turn almond waste into ethanol.

**Figure:**
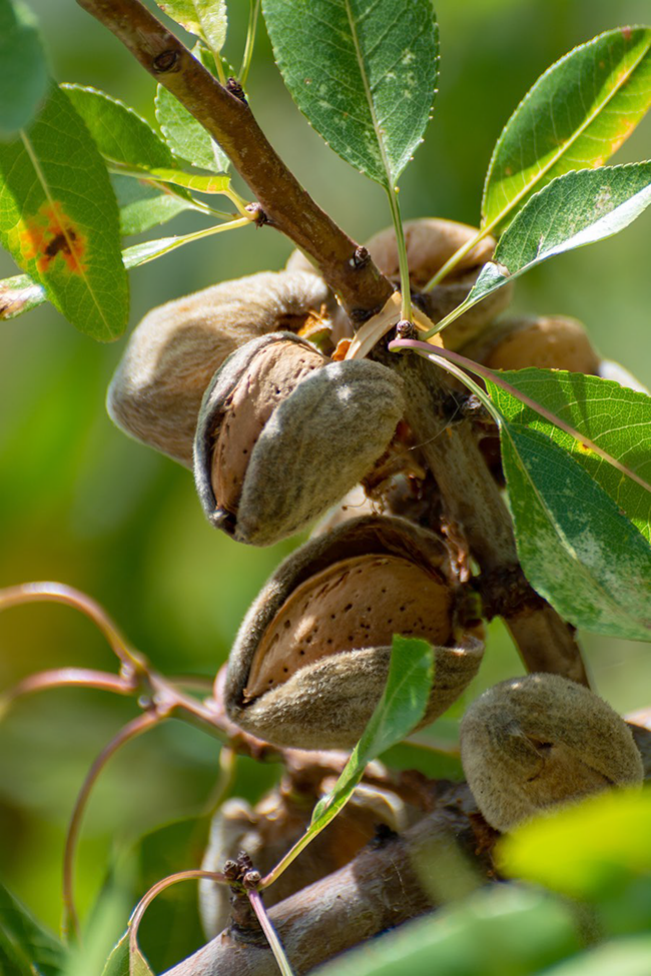
Almond nuts are surrounded by a fuzzy, fruit-like hull over a wood-like shell. Credit: Shutterstock.

Technically, the answer was yes. But the team concluded that making ethanol from almond waste was not cost competitive with making ethanol from corn.

Due to the shells’ similarity to wood, the team’s next thought was to turn them into charcoal briquettes. This entailed grinding and then partially burning the ground almond shells by heating them to about 300 °C in the absence of oxygen, a process called torrefaction. The idea was to make plant-based charcoal briquettes to displace some of the petroleum-based coal that was once widely used in California to generate electricity. While almond shells made good briquettes, the market for them disappeared when the state switched from coal to cleaner sources of energy.

Then the team discovered a greener use for torrefied almond shells. “Almond charcoal is a good substitute for carbon black, which is used to make recycled plastic uniform and more appealing,” Orts says. Carbon black, which resembles soot, is typically made by burning coal. “We thought, ‘Wouldn’t it be nicer to produce it locally from sustainable carbon?’ ”

Untreated almond shells contain too much cellulose and hemicellulose, which are hydrophilic, to blend with plastic, which is hydrophobic. Torrefaction degrades these polysaccharides, leaving an ink-black, carbon-rich substance. The USDA team showed that torrefied almond shells mix well with recycled plastic and turn it from what Orts calls an “ugly gray” to a deep black.

Torrefied almond shells also enhance recycled plastic’s mechanical properties, according to a USDA study. Recycling plastic involves melting and grinding, which breaks the polymer chains and weakens the material. Torrefied shells restrict the mobility of these chains and “make the plastic stiffer,” says USDA chemical engineer Zach McCaffrey, who led the study. They also make recycled plastic more heat stable.

Orts and his team partnered with a California manufacturer called TranPak; the partners began to produce recycled plastic pallets that, like the prototype in the USDA facility, contain 15% torrefied almond shells. The pandemic interrupted this collaboration, but Orts hopes to jump-start it again soon.

Walking around the USDA facility, one sees that Orts is not alone in exhibiting the unexpected. For example, chemist Colleen McMahan appears to have dumped a car tire outside her office door. McMahan, now acting leader of the bioproducts research team, spent a dozen years developing tire compounds at Goodyear Tire & Rubber Company before joining the USDA. She says that tires can be more sustainable and that this prototype is proof.

**Figure:**
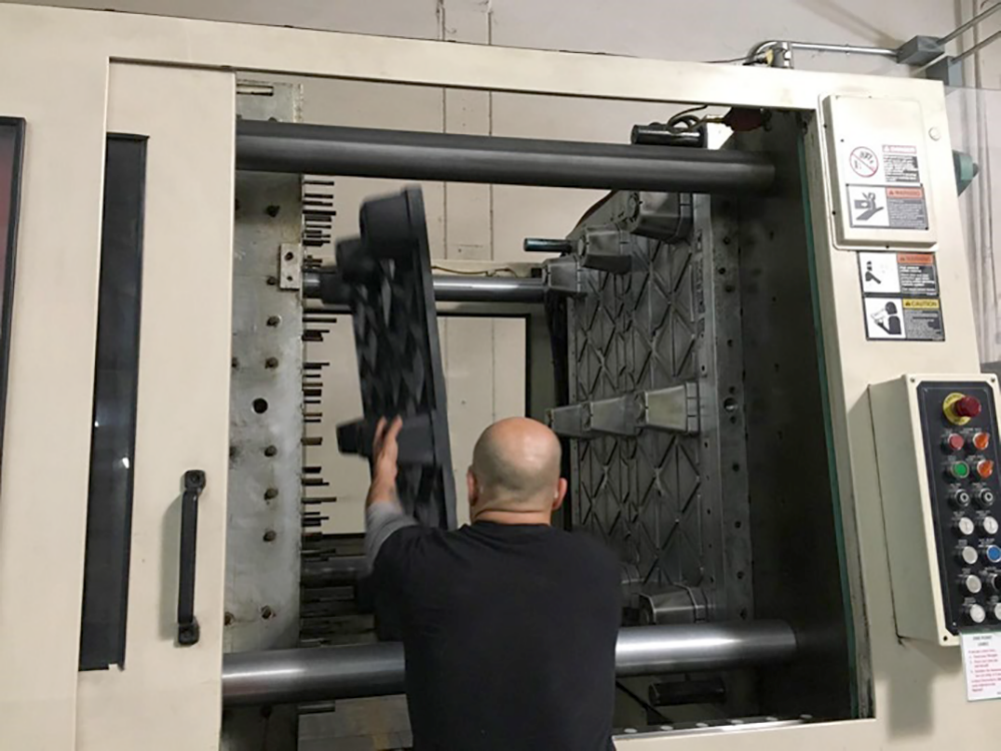
TranPak worker making recycled plastic pallets that contain torrefied almond shells in Fresno, California Credit: Delilah F. Wood.

The tire contains rubber not from rubber tree plantations in the tropics but from guayule, a shrub native to and currently being farmed in the southwestern US. Orts hopes the next iteration of this tire will also feature torrefied almond shells as a sustainable alternative to the usual carbon black.

Manufacturers add carbon black to tires to reinforce their rubber—and without it, tires would be white. “The biggest use of carbon black in the world is making tires. Companies need billions of pounds,” Orts says.

Tires are roughly one part carbon black to two parts rubber, McMahan says. A study she coauthored showed that torrefied almond shells mix well with rubber and could replace some of the coal-derived carbon black while preserving the desired mechanical properties of the rubber.

While the USDA sees high potential in almond shells—which go to low-value uses today—the agency’s researchers are also investigating higher-value uses for almond hulls as fewer hulls are being fed to cattle. Near the onset of their pursuit, the researchers squeezed the sugary juice out of hulls and sent the resulting syrup to entomologist Gloria DeGrandi-Hoffman, research lead at the USDA’s Carl Hayden Bee Research Center.

The almond industry depends on honeybee hives that are trucked in to pollinate orchards in early spring. Beekeepers must maintain these hives over the winter to ensure the bees are raring to go as soon as almond blossoms appear. During the colder months, when natural flower nectar is in low supply, keepers feed high-fructose corn syrup to their tiny charges, but Orts says that bees “don’t thrive on it.” The bioproducts researchers wondered if high-fructose corn syrup could be replaced with almond hull sugars, which would have the added appeal of using almond waste to grow more of the nuts.

DeGrandi-Hoffman’s team found that bees slurp up the sugar from almond hulls with gusto—their stingers flicking up in the air as they dig their heads into a tiny pot of syrup. But the project was ultimately a bust. “The sugar solutions were highly toxic to bees,” DeGrandi-Hoffman says in an email. She speculates that this was because of the tannins in the hull syrup.

From the literal remnants of this project, however, the bioproducts team found another promising application. After the juice is extracted, the fiber-rich hulls can absorb a lot of water; that reminded the researchers of sphagnum moss, spongy plant matter that mushroom producers use to keep their growth media moist. Sphagnum moss is marketed as renewable and sustainable, but it is harvested from bogs outside the US and then shipped into the country, which adds to its carbon footprint, Orts says. And even though it is renewable in the broad sense, it grows back slowly, so it is not as sustainable as it might seem, he adds. “We want a renewable, local source.”

The bioproducts team worked with Premier Mushrooms, a California-based company, to test various proportions of spent hulls and sphagnum moss; mushrooms flourished in a 50–50 mix. The project came to a halt when Premier went out of business during the pandemic, but Orts hopes to continue it with a new partner.

Given the hulls’ similarity to peaches, the Almond Board wants to use hulls in human food. The board gave ground almond hulls—tannins and all—to Mattson, a company that develops foods and beverages. Mattson prepared a range of hull-based edibles for taste tests. Most of them were flops. The beer was “too bitter even for IPA lovers,” Orts says. And Veenstra at the Almond Board describes the bread as “dense and gritty,”

But the performance bar, which was 15% hulls, was a hit. “It was fruity, sweet, and bitter” but pleasantly so, Veenstra says. So after decades of feeding hulls to livestock, the board is now asking the U.S. Food and Drug Administration for approval to feed hulls to people.

Some new uses are even driven by growers, who are taking the circular approach of repurposing their almond byproducts in their own orchards.

Almond grower Connor Wagner was inspired after hearing Orts present at a 2019 Almond Board meeting at the University of California, Davis. With the USDA bioproducts team and rubber manufacturers, the grower used torrefied almond shells to replace some of the carbon black in the rubber pads attached to almond-harvesting machines. These devices clamp onto tree trunks and shake them so vigorously that almonds rain down; the rubber pads affixed to the machines’ arms help protect the bark.

Christopher Simmons, a biological systems engineer at UC Davis, is also testing a new way to reinject almond waste into farms. He wants to use almond hulls and shells to rid almond orchards of nematode infestations. These microscopic roundworms eat roots, stunting plants; young trees are particularly vulnerable. Before planting new orchards, almond growers kill nematodes by fumigating the soil with pesticides.

Simmons began his almond orchard study after presenting his work on a fumigation alternative—called biosolarization—during an Almond Board visit to his campus in 2017. The technique uses a combination of sun and biomass, including almond byproducts, to control pests and pathogens in soil.

Simmons recalls almond grower Rory Crowley saying that he favored a pesticide-free approach to replanting trees partly because the orchard was next to a daycare center.

The pilot study, conducted on 3.6 ha of land Crowley farmed, entailed covering the soil with milled almond hulls and shells, getting the mixture wet, and covering it with clear plastic. Under the California sun, the plastic locked heat in the soil, and it cut off oxygen. The fiber-rich almond waste provided nutrients for microbes so that all together, biosolarization created ideal conditions for anaerobic bacteria to thrive. These bacteria produced acetic acid and other compounds that disinfested the soil.

After 9 days of biosolarization, there was a “complete kill” of the nematodes the researchers tested for in the study. Unpublished work by others suggests that “biosolarization could be cost competitive or even cheaper than fumigation,” Simmons says. The existing analysis likely underestimates the potential cost savings, he adds, because it does not account for the fact that nutrients from almond waste could save growers money by reducing the need for fertilizer.

Widespread adoption of new uses for almond hulls and shells will ultimately come down to economics, says Gemperle, the farmer and Almond Board member. “For some of these things, the science is there. But is it cost effective for the grower?”

## Robin Meadows is a freelance contributor to

Chemical & Engineering News, *the independent news outlet of the American Chemical Society*.

